# Diethyl 3,3′-(phenyl­methyl­ene)bis­(1*H*-indole-2-carboxyl­ate)

**DOI:** 10.1107/S1600536812036239

**Published:** 2012-08-25

**Authors:** Hong-Shun Sun, Yu-Long Li, Ning Xu, Hong Xu, Ji-Dong Zhang

**Affiliations:** a Chemical Engineering Department, Nanjing College of Chemical Technology, Nanjing 210048, People’s Republic of China

## Abstract

In the title compound, C_29_H_26_N_2_O_4_, the benzene ring is twisted by 73.5 (5) and 84.9 (3)° with respect to the mean planes of the two indole ring systems; the mean planes of the indole ring systems are oriented at a dihedral angle of 82.0 (5)°. In the crystal, mol­ecules are linked by pairs of N—H⋯O hydrogen bonds into chains.

## Related literature

For applications of indole derivatives, see: Poter *et al.* (1977 ▶); Sundberg (1996 ▶); Chang *et al.* (1999 ▶); Ge *et al.* (1999 ▶); Ni (2008 ▶).
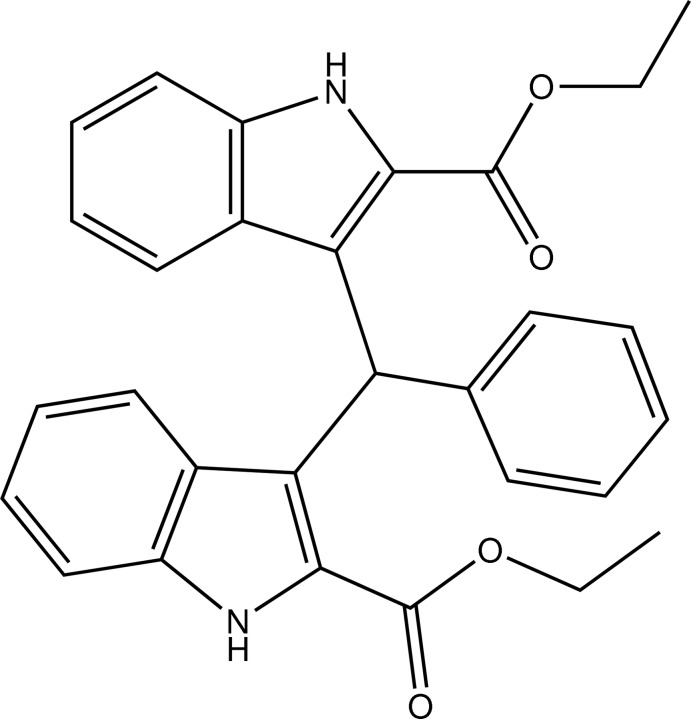


## Experimental

### 

#### Crystal data

C_29_H_26_N_2_O_4_
*M* *_r_* = 466.52
Triclinic, 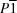

*a* = 8.7340 (17) Å
*b* = 9.871 (2) Å
*c* = 15.000 (3) Å
α = 76.14 (3)°
β = 83.91 (3)°
γ = 83.09 (3)°
*V* = 1242.4 (4) Å^3^
*Z* = 2
Mo *K*α radiation
μ = 0.08 mm^−1^
*T* = 293 K
0.30 × 0.20 × 0.10 mm

#### Data collection

Enraf–Nonius CAD-4 diffractometer
4879 measured reflections
4557 independent reflections
2813 reflections with *I* > 2σ(*I*)
*R*_int_ = 0.032
3 standard reflections every 200 reflections intensity decay: 1%

#### Refinement

*R*[*F* ^2^ > 2σ(*F* ^2^)] = 0.059
*wR*(*F* ^2^) = 0.144
*S* = 1.00
4557 reflections
316 parameters
2 restraints
H-atom parameters constrained
Δρ_max_ = 0.33 e Å^−3^
Δρ_min_ = −0.24 e Å^−3^

### 

Data collection: *CAD-4 EXPRESS* (Enraf–Nonius, 1994 ▶); cell refinement: *CAD-4 EXPRESS*; data reduction: *XCAD4* (Harms & Wocadlo, 1995 ▶); program(s) used to solve structure: *SHELXTL* (Sheldrick, 2008 ▶); program(s) used to refine structure: *SHELXTL*; molecular graphics: *SHELXTL*; software used to prepare material for publication: *SHELXTL*.

## Supplementary Material

Crystal structure: contains datablock(s) I, global. DOI: 10.1107/S1600536812036239/xu5612sup1.cif

Structure factors: contains datablock(s) I. DOI: 10.1107/S1600536812036239/xu5612Isup2.hkl

Supplementary material file. DOI: 10.1107/S1600536812036239/xu5612Isup3.cml

Additional supplementary materials:  crystallographic information; 3D view; checkCIF report

## Figures and Tables

**Table 1:** Hydrogen-bond geometry (Å, °)

*D*—H⋯*A*	*D*—H	H⋯*A*	*D*⋯*A*	*D*—H⋯*A*
N1—H1*A*⋯O2^i^	0.86	2.21	2.955 (3)	144
N2—H2*A*⋯O4^ii^	0.86	2.07	2.880 (3)	157

Symmetry codes: (i) 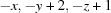; (ii) 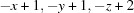.

**Fig. 1.**
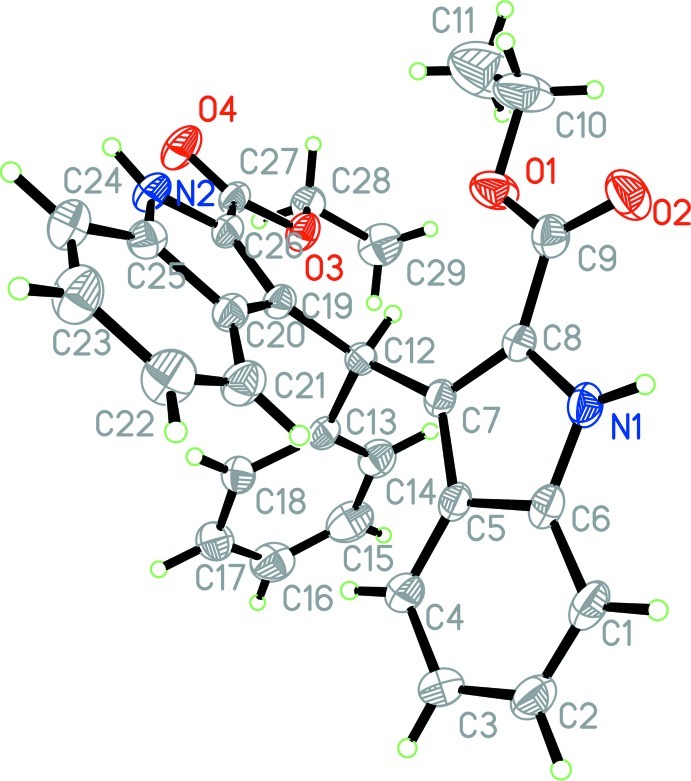
The molecular structure of (I), showing the atom-numbering scheme and displacement ellipsoids at the 30% probability level.

**Fig. 2.**
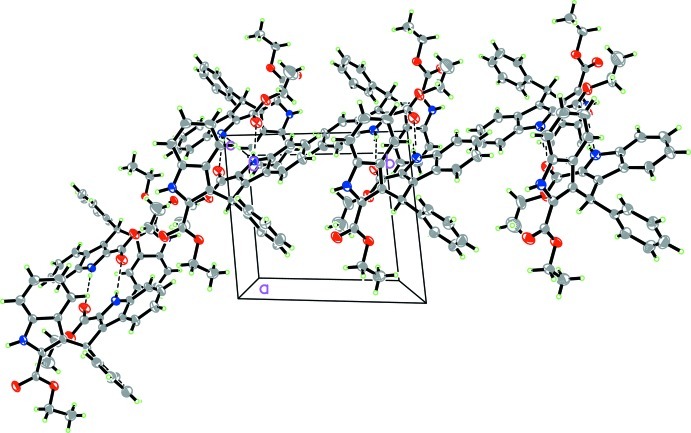
A packing diagram of (I). Intermolecular hydrogen bonds are shown as dashed lines.

## supplementary crystallographic information

### Comment

Indole derivatives are found abundantly in a variety of natural plants and exhibit various physiological properties (Poter *et al.*, 1977; Sundberg, 1996). Among them, bis-indolymethane derivatives are found to be kinds of potentially bioactive compounds (Chang *et al.*, 1999; Ge *et al.*, 1999). In recent years, the synthesis and application of bis-indolymethane derivatives have been widely studied. The title compound is one of bis-indolymethane derivatives as a precursor for MRI Contrast Agents (Ni, 2008). We report here its crystal structure.

The molecular structure of the title compound is shown in Fig. 1. The benzene ring is twisted to the two indole rings with the dihedral angles of 73.5 (5) and 84.9 (3)°, respectively. Two indole rings make a dihedral angle of 82.0 (5)° to each other.

As shown in Figure 2, the molecules are linked by paired N—H···O hydrogen bonds into dimers in the crystal lattice. The structural parameters for the intermolecular hydrogen bonds resulting in the formation of dimers are given in Table 1.

### Experimental

Ethyl indole-2-carboxylate (18.9 g, 100 mmol) was dissolved in 200 ml ethanol; commercially available benzaldehyde (5.3 g, 50 mmol) was added and the mixture was heated to reflux temperature. Concentrated HCl (3.7 ml) was added and the reaction was left for 1 h. After cooling the white product was filtered off and washed thoroughly with ethanol. The reaction can be followed by TLC (CHCl_3_:hexane = 1:1). Yield was 90%. Crystals of the title compound suitable for X-ray analysis were obtained by slow evaporation of an ethanol solution.

### Refinement

H atoms were positioned geometrically, with N— H = 0.86 Å and C—H = 0.93, 0.96, 0.97 and 0.98 Å for aromatic, methyl, methylene and methine H, respectively, and constrained to ride on their parent atoms, with *U*_iso_(H) = xU_eq_(C,N), where *x* = 1.5 for methyl H, and *x* = 1.2 for all other H atoms.

### Crystal data

**Table:**

C_29_H_26_N_2_O_4_	*Z* = 2
*M**_r_* = 466.52	*F*(000) = 492
Triclinic, *P*1	*D*_x_ = 1.247 Mg m^−^^3^
Hall symbol: -P 1	Mo *K*α radiation, λ = 0.71073 Å
*a* = 8.7340 (17) Å	Cell parameters from 25 reflections
*b* = 9.871 (2) Å	θ = 9–13°
*c* = 15.000 (3) Å	µ = 0.08 mm^−^^1^
α = 76.14 (3)°	*T* = 293 K
β = 83.91 (3)°	Block, colorless
γ = 83.09 (3)°	0.30 × 0.20 × 0.10 mm
*V* = 1242.4 (4) Å^3^	

### Data collection

**Table:**

Enraf–Nonius CAD-4 diffractometer	*R*_int_ = 0.032
Radiation source: fine-focus sealed tube	θ_max_ = 25.4°, θ_min_ = 1.4°
Graphite monochromator	*h* = 0→10
ω/2θ scans	*k* = −11→11
4879 measured reflections	*l* = −17→18
4557 independent reflections	3 standard reflections every 200 reflections
2813 reflections with *I* > 2σ(*I*)	intensity decay: 1%

### Refinement

**Table:**

Refinement on *F*^2^	Primary atom site location: structure-invariant direct methods
Least-squares matrix: full	Secondary atom site location: difference Fourier map
*R*[*F*^2^ > 2σ(*F*^2^)] = 0.059	Hydrogen site location: inferred from neighbouring sites
*wR*(*F*^2^) = 0.144	H-atom parameters constrained
*S* = 1.00	*w* = 1/[σ^2^(*F*_o_^2^) + (0.068*P*)^2^] where *P* = (*F*_o_^2^ + 2*F*_c_^2^)/3
4557 reflections	(Δ/σ)_max_ < 0.001
316 parameters	Δρ_max_ = 0.33 e Å^−^^3^
2 restraints	Δρ_min_ = −0.24 e Å^−^^3^

### Special details

**Table:**

Geometry. All e.s.d.'s (except the e.s.d. in the dihedral angle between two l.s. planes) are estimated using the full covariance matrix. The cell e.s.d.'s are taken into account individually in the estimation of e.s.d.'s in distances, angles and torsion angles; correlations between e.s.d.'s in cell parameters are only used when they are defined by crystal symmetry. An approximate (isotropic) treatment of cell e.s.d.'s is used for estimating e.s.d.'s involving l.s. planes.
Refinement. Refinement of *F*^2^ against ALL reflections. The weighted *R*-factor *wR* and goodness of fit *S* are based on *F*^2^, conventional *R*-factors *R* are based on *F*, with *F* set to zero for negative *F*^2^. The threshold expression of *F*^2^ > σ(*F*^2^) is used only for calculating *R*-factors(gt) *etc*. and is not relevant to the choice of reflections for refinement. *R*-factors based on *F*^2^ are statistically about twice as large as those based on *F*, and *R*- factors based on ALL data will be even larger.

### Fractional atomic coordinates and isotropic or equivalent isotropic displacement parameters (Å^2^)

**Table:**

	*x*	*y*	*z*	*U*_iso_*/*U*_eq_	
N1	0.1091 (2)	1.1102 (2)	0.57403 (13)	0.0464 (5)	
H1A	0.0562	1.1030	0.5305	0.056*	
O1	0.3652 (2)	0.79594 (18)	0.61548 (14)	0.0671 (6)	
C1	0.0036 (3)	1.3484 (3)	0.59245 (18)	0.0543 (7)	
H1B	−0.0653	1.3654	0.5469	0.065*	
N2	0.3177 (2)	0.6564 (2)	0.93548 (13)	0.0455 (5)	
H2A	0.3453	0.5760	0.9700	0.055*	
O2	0.1783 (2)	0.8636 (2)	0.51907 (13)	0.0684 (6)	
C2	0.0155 (3)	1.4450 (3)	0.64135 (19)	0.0622 (8)	
H2B	−0.0449	1.5305	0.6283	0.075*	
O3	0.65528 (18)	0.75379 (17)	0.78745 (11)	0.0501 (5)	
C3	0.1166 (3)	1.4189 (3)	0.71086 (19)	0.0617 (8)	
H3A	0.1214	1.4872	0.7435	0.074*	
O4	0.6289 (2)	0.57275 (18)	0.90776 (13)	0.0650 (6)	
C4	0.2091 (3)	1.2949 (3)	0.73230 (18)	0.0556 (7)	
H4A	0.2753	1.2789	0.7791	0.067*	
C5	0.2020 (3)	1.1927 (2)	0.68248 (15)	0.0408 (6)	
C6	0.0979 (3)	1.2226 (3)	0.61280 (16)	0.0451 (6)	
C7	0.2770 (3)	1.0539 (2)	0.68458 (15)	0.0384 (6)	
C8	0.2186 (3)	1.0099 (2)	0.61581 (15)	0.0402 (6)	
C9	0.2502 (3)	0.8841 (3)	0.57918 (18)	0.0491 (6)	
C10	0.4101 (4)	0.6726 (3)	0.5781 (3)	0.0934 (12)	
H10A	0.3348	0.6054	0.6016	0.112*	
H10B	0.4081	0.6996	0.5116	0.112*	
C11	0.5551 (5)	0.6089 (5)	0.5996 (4)	0.1460 (19)	
H11A	0.5781	0.5285	0.5733	0.219*	
H11B	0.5575	0.5800	0.6653	0.219*	
H11C	0.6308	0.6740	0.5752	0.219*	
C12	0.3962 (3)	0.9705 (2)	0.74867 (15)	0.0373 (6)	
H12A	0.4708	0.9217	0.7106	0.045*	
C13	0.4886 (3)	1.0644 (2)	0.78505 (16)	0.0395 (6)	
C14	0.6008 (3)	1.1351 (3)	0.72766 (19)	0.0568 (7)	
H14A	0.6244	1.1202	0.6686	0.068*	
C15	0.6790 (4)	1.2282 (3)	0.7569 (3)	0.0764 (9)	
H15A	0.7541	1.2759	0.7170	0.092*	
C16	0.6473 (4)	1.2511 (3)	0.8438 (2)	0.0710 (9)	
H16A	0.6991	1.3154	0.8624	0.085*	
C17	0.5397 (3)	1.1794 (3)	0.9029 (2)	0.0611 (8)	
H17A	0.5190	1.1934	0.9623	0.073*	
C18	0.4605 (3)	1.0849 (3)	0.87424 (17)	0.0478 (6)	
H18A	0.3882	1.0350	0.9151	0.057*	
C19	0.3294 (3)	0.8562 (2)	0.82476 (15)	0.0374 (6)	
C20	0.1766 (3)	0.8539 (2)	0.87173 (15)	0.0395 (6)	
C21	0.0399 (3)	0.9474 (3)	0.86615 (18)	0.0511 (7)	
H21A	0.0363	1.0322	0.8226	0.061*	
C22	−0.0862 (3)	0.9119 (3)	0.92517 (19)	0.0619 (8)	
H22A	−0.1757	0.9738	0.9214	0.074*	
C23	−0.0847 (3)	0.7852 (3)	0.9912 (2)	0.0632 (8)	
H23A	−0.1734	0.7642	1.0298	0.076*	
C24	0.0448 (3)	0.6912 (3)	1.00012 (17)	0.0527 (7)	
H24A	0.0464	0.6071	1.0443	0.063*	
C25	0.1743 (3)	0.7277 (2)	0.93981 (16)	0.0426 (6)	
C26	0.4107 (3)	0.7330 (2)	0.86766 (15)	0.0388 (6)	
C27	0.5734 (3)	0.6772 (2)	0.85723 (16)	0.0402 (6)	
C28	0.8177 (3)	0.7041 (3)	0.77442 (18)	0.0553 (7)	
H28A	0.8282	0.6102	0.7638	0.066*	
H28B	0.8701	0.7019	0.8287	0.066*	
C29	0.8863 (3)	0.8028 (3)	0.6932 (2)	0.0802 (10)	
H29A	0.9940	0.7727	0.6825	0.120*	
H29B	0.8759	0.8952	0.7048	0.120*	
H29C	0.8335	0.8044	0.6400	0.120*	

### Atomic displacement parameters (Å^2^)

**Table:**

	*U*^11^	*U*^22^	*U*^33^	*U*^12^	*U*^13^	*U*^23^
N1	0.0488 (13)	0.0511 (13)	0.0378 (11)	0.0003 (10)	−0.0143 (10)	−0.0049 (10)
O1	0.0723 (14)	0.0525 (12)	0.0859 (14)	0.0166 (10)	−0.0364 (11)	−0.0322 (10)
C1	0.0510 (16)	0.0538 (17)	0.0462 (15)	0.0093 (13)	−0.0099 (13)	0.0071 (13)
N2	0.0469 (13)	0.0359 (11)	0.0461 (12)	0.0014 (10)	−0.0069 (10)	0.0039 (9)
O2	0.0705 (13)	0.0795 (14)	0.0662 (13)	0.0077 (11)	−0.0280 (11)	−0.0355 (11)
C2	0.067 (2)	0.0520 (17)	0.0555 (18)	0.0173 (15)	−0.0037 (15)	−0.0005 (15)
O3	0.0440 (10)	0.0501 (10)	0.0468 (10)	0.0059 (8)	−0.0009 (8)	0.0007 (8)
C3	0.076 (2)	0.0451 (16)	0.0604 (18)	0.0116 (15)	−0.0099 (16)	−0.0114 (13)
O4	0.0580 (12)	0.0507 (11)	0.0675 (12)	0.0134 (9)	−0.0037 (10)	0.0132 (10)
C4	0.0693 (19)	0.0456 (16)	0.0508 (16)	0.0055 (14)	−0.0184 (14)	−0.0086 (13)
C5	0.0446 (14)	0.0399 (14)	0.0320 (13)	0.0002 (11)	−0.0026 (11)	0.0008 (11)
C6	0.0468 (15)	0.0456 (15)	0.0356 (14)	−0.0011 (12)	−0.0037 (11)	0.0032 (11)
C7	0.0397 (13)	0.0400 (13)	0.0317 (12)	0.0015 (11)	−0.0049 (11)	−0.0030 (10)
C8	0.0442 (14)	0.0384 (13)	0.0361 (13)	0.0003 (11)	−0.0100 (11)	−0.0040 (11)
C9	0.0489 (16)	0.0536 (16)	0.0442 (15)	−0.0016 (13)	−0.0104 (13)	−0.0088 (13)
C10	0.098 (3)	0.060 (2)	0.138 (3)	0.0167 (19)	−0.046 (2)	−0.050 (2)
C11	0.123 (4)	0.135 (4)	0.200 (5)	0.021 (3)	−0.042 (4)	−0.082 (4)
C12	0.0407 (14)	0.0348 (13)	0.0353 (13)	0.0057 (11)	−0.0102 (11)	−0.0073 (10)
C13	0.0398 (14)	0.0362 (13)	0.0390 (14)	0.0037 (11)	−0.0102 (11)	−0.0026 (11)
C14	0.0574 (17)	0.0537 (17)	0.0561 (17)	−0.0123 (14)	0.0014 (14)	−0.0055 (14)
C15	0.069 (2)	0.0589 (19)	0.097 (3)	−0.0266 (17)	0.0001 (19)	−0.0023 (18)
C16	0.074 (2)	0.0601 (19)	0.085 (2)	−0.0156 (17)	−0.0234 (19)	−0.0172 (18)
C17	0.074 (2)	0.0598 (18)	0.0549 (17)	−0.0035 (16)	−0.0240 (16)	−0.0178 (15)
C18	0.0524 (16)	0.0494 (15)	0.0412 (15)	−0.0021 (13)	−0.0084 (12)	−0.0090 (12)
C19	0.0405 (14)	0.0338 (13)	0.0363 (13)	0.0004 (11)	−0.0105 (11)	−0.0036 (10)
C20	0.0413 (14)	0.0376 (13)	0.0379 (13)	−0.0032 (11)	−0.0069 (11)	−0.0042 (10)
C21	0.0479 (16)	0.0469 (15)	0.0527 (16)	0.0056 (13)	−0.0080 (13)	−0.0034 (12)
C22	0.0407 (16)	0.0669 (19)	0.070 (2)	0.0074 (14)	0.0033 (15)	−0.0103 (16)
C23	0.0467 (17)	0.071 (2)	0.0633 (19)	−0.0068 (15)	0.0103 (14)	−0.0054 (16)
C24	0.0485 (16)	0.0557 (17)	0.0483 (16)	−0.0087 (14)	−0.0017 (13)	−0.0004 (13)
C25	0.0413 (14)	0.0420 (14)	0.0442 (14)	−0.0024 (12)	−0.0085 (12)	−0.0080 (11)
C26	0.0444 (14)	0.0343 (13)	0.0343 (13)	0.0017 (11)	−0.0066 (11)	−0.0023 (10)
C27	0.0447 (15)	0.0333 (13)	0.0382 (13)	0.0012 (11)	−0.0054 (12)	−0.0014 (11)
C28	0.0501 (17)	0.0554 (17)	0.0543 (17)	0.0047 (13)	0.0025 (13)	−0.0084 (13)
C29	0.065 (2)	0.082 (2)	0.088 (2)	−0.0115 (18)	0.0150 (18)	−0.0142 (19)

### Geometric parameters (Å, º)

**Table:**

N1—C6	1.361 (3)	C12—C13	1.524 (3)
N1—C8	1.375 (3)	C12—H12A	0.9800
N1—H1A	0.8600	C13—C14	1.374 (3)
O1—C9	1.320 (3)	C13—C18	1.392 (3)
O1—C10	1.455 (3)	C14—C15	1.382 (4)
C1—C2	1.354 (4)	C14—H14A	0.9300
C1—C6	1.393 (3)	C15—C16	1.370 (4)
C1—H1B	0.9300	C15—H15A	0.9300
N2—C25	1.364 (3)	C16—C17	1.362 (4)
N2—C26	1.368 (3)	C16—H16A	0.9300
N2—H2A	0.8600	C17—C18	1.394 (3)
O2—C9	1.219 (3)	C17—H17A	0.9300
C2—C3	1.394 (4)	C18—H18A	0.9300
C2—H2B	0.9300	C19—C26	1.382 (3)
O3—C27	1.331 (3)	C19—C20	1.442 (3)
O3—C28	1.453 (3)	C20—C25	1.410 (3)
C3—C4	1.372 (3)	C20—C21	1.415 (3)
C3—H3A	0.9300	C21—C22	1.363 (3)
O4—C27	1.207 (3)	C21—H21A	0.9300
C4—C5	1.402 (3)	C22—C23	1.397 (4)
C4—H4A	0.9300	C22—H22A	0.9300
C5—C6	1.413 (3)	C23—C24	1.371 (4)
C5—C7	1.440 (3)	C23—H23A	0.9300
C7—C8	1.375 (3)	C24—C25	1.397 (3)
C7—C12	1.520 (3)	C24—H24A	0.9300
C8—C9	1.460 (3)	C26—C27	1.467 (3)
C10—C11	1.379 (4)	C28—C29	1.486 (4)
C10—H10A	0.9700	C28—H28A	0.9700
C10—H10B	0.9700	C28—H28B	0.9700
C11—H11A	0.9600	C29—H29A	0.9600
C11—H11B	0.9600	C29—H29B	0.9600
C11—H11C	0.9600	C29—H29C	0.9600
C12—C19	1.522 (3)		
			
C6—N1—C8	108.91 (19)	C18—C13—C12	122.3 (2)
C6—N1—H1A	125.5	C13—C14—C15	120.6 (3)
C8—N1—H1A	125.5	C13—C14—H14A	119.7
C9—O1—C10	117.3 (2)	C15—C14—H14A	119.7
C2—C1—C6	117.7 (3)	C16—C15—C14	120.8 (3)
C2—C1—H1B	121.2	C16—C15—H15A	119.6
C6—C1—H1B	121.2	C14—C15—H15A	119.6
C25—N2—C26	109.28 (19)	C17—C16—C15	119.7 (3)
C25—N2—H2A	125.4	C17—C16—H16A	120.2
C26—N2—H2A	125.4	C15—C16—H16A	120.2
C1—C2—C3	121.4 (3)	C16—C17—C18	120.0 (3)
C1—C2—H2B	119.3	C16—C17—H17A	120.0
C3—C2—H2B	119.3	C18—C17—H17A	120.0
C27—O3—C28	116.10 (19)	C13—C18—C17	120.6 (3)
C4—C3—C2	121.6 (3)	C13—C18—H18A	119.7
C4—C3—H3A	119.2	C17—C18—H18A	119.7
C2—C3—H3A	119.2	C26—C19—C20	105.12 (19)
C3—C4—C5	119.0 (2)	C26—C19—C12	125.5 (2)
C3—C4—H4A	120.5	C20—C19—C12	129.27 (19)
C5—C4—H4A	120.5	C25—C20—C21	117.3 (2)
C4—C5—C6	117.8 (2)	C25—C20—C19	107.5 (2)
C4—C5—C7	135.3 (2)	C21—C20—C19	135.2 (2)
C6—C5—C7	106.9 (2)	C22—C21—C20	119.4 (2)
N1—C6—C1	129.3 (2)	C22—C21—H21A	120.3
N1—C6—C5	108.1 (2)	C20—C21—H21A	120.3
C1—C6—C5	122.6 (2)	C21—C22—C23	121.8 (3)
C8—C7—C5	105.8 (2)	C21—C22—H22A	119.1
C8—C7—C12	126.1 (2)	C23—C22—H22A	119.1
C5—C7—C12	128.1 (2)	C24—C23—C22	121.3 (3)
N1—C8—C7	110.2 (2)	C24—C23—H23A	119.3
N1—C8—C9	116.0 (2)	C22—C23—H23A	119.3
C7—C8—C9	133.8 (2)	C23—C24—C25	116.9 (2)
O2—C9—O1	122.9 (3)	C23—C24—H24A	121.5
O2—C9—C8	122.9 (2)	C25—C24—H24A	121.5
O1—C9—C8	114.2 (2)	N2—C25—C24	129.0 (2)
C11—C10—O1	113.3 (3)	N2—C25—C20	107.6 (2)
C11—C10—H10A	108.9	C24—C25—C20	123.3 (2)
O1—C10—H10A	108.9	N2—C26—C19	110.4 (2)
C11—C10—H10B	108.9	N2—C26—C27	117.0 (2)
O1—C10—H10B	108.9	C19—C26—C27	132.5 (2)
H10A—C10—H10B	107.7	O4—C27—O3	122.8 (2)
C10—C11—H11A	109.5	O4—C27—C26	123.4 (2)
C10—C11—H11B	109.5	O3—C27—C26	113.7 (2)
H11A—C11—H11B	109.5	O3—C28—C29	107.3 (2)
C10—C11—H11C	109.5	O3—C28—H28A	110.2
H11A—C11—H11C	109.5	C29—C28—H28A	110.2
H11B—C11—H11C	109.5	O3—C28—H28B	110.2
C7—C12—C19	113.36 (18)	C29—C28—H28B	110.2
C7—C12—C13	112.51 (18)	H28A—C28—H28B	108.5
C19—C12—C13	112.78 (18)	C28—C29—H29A	109.5
C7—C12—H12A	105.8	C28—C29—H29B	109.5
C19—C12—H12A	105.8	H29A—C29—H29B	109.5
C13—C12—H12A	105.8	C28—C29—H29C	109.5
C14—C13—C18	118.2 (2)	H29A—C29—H29C	109.5
C14—C13—C12	119.4 (2)	H29B—C29—H29C	109.5
			
C6—C1—C2—C3	−1.2 (4)	C13—C14—C15—C16	0.5 (5)
C1—C2—C3—C4	0.5 (4)	C14—C15—C16—C17	1.3 (5)
C2—C3—C4—C5	0.4 (4)	C15—C16—C17—C18	−1.1 (4)
C3—C4—C5—C6	−0.6 (4)	C14—C13—C18—C17	2.8 (4)
C3—C4—C5—C7	−179.5 (3)	C12—C13—C18—C17	−175.3 (2)
C8—N1—C6—C1	179.6 (2)	C16—C17—C18—C13	−1.0 (4)
C8—N1—C6—C5	−0.8 (3)	C7—C12—C19—C26	152.1 (2)
C2—C1—C6—N1	−179.6 (2)	C13—C12—C19—C26	−78.6 (3)
C2—C1—C6—C5	0.9 (4)	C7—C12—C19—C20	−32.3 (3)
C4—C5—C6—N1	−179.6 (2)	C13—C12—C19—C20	97.0 (3)
C7—C5—C6—N1	−0.5 (3)	C26—C19—C20—C25	−0.8 (2)
C4—C5—C6—C1	0.0 (4)	C12—C19—C20—C25	−177.0 (2)
C7—C5—C6—C1	179.1 (2)	C26—C19—C20—C21	176.8 (3)
C4—C5—C7—C8	−179.5 (3)	C12—C19—C20—C21	0.5 (4)
C6—C5—C7—C8	1.6 (2)	C25—C20—C21—C22	−0.5 (4)
C4—C5—C7—C12	0.6 (4)	C19—C20—C21—C22	−177.8 (3)
C6—C5—C7—C12	−178.3 (2)	C20—C21—C22—C23	−0.2 (4)
C6—N1—C8—C7	1.9 (3)	C21—C22—C23—C24	0.7 (5)
C6—N1—C8—C9	−177.3 (2)	C22—C23—C24—C25	−0.5 (4)
C5—C7—C8—N1	−2.2 (3)	C26—N2—C25—C24	−178.0 (2)
C12—C7—C8—N1	177.8 (2)	C26—N2—C25—C20	0.3 (3)
C5—C7—C8—C9	176.9 (3)	C23—C24—C25—N2	177.9 (2)
C12—C7—C8—C9	−3.2 (4)	C23—C24—C25—C20	−0.2 (4)
C10—O1—C9—O2	2.7 (4)	C21—C20—C25—N2	−177.8 (2)
C10—O1—C9—C8	−176.1 (2)	C19—C20—C25—N2	0.3 (3)
N1—C8—C9—O2	−4.4 (4)	C21—C20—C25—C24	0.7 (4)
C7—C8—C9—O2	176.7 (3)	C19—C20—C25—C24	178.7 (2)
N1—C8—C9—O1	174.4 (2)	C25—N2—C26—C19	−0.8 (3)
C7—C8—C9—O1	−4.6 (4)	C25—N2—C26—C27	175.8 (2)
C9—O1—C10—C11	162.2 (4)	C20—C19—C26—N2	1.0 (3)
C8—C7—C12—C19	−76.6 (3)	C12—C19—C26—N2	177.4 (2)
C5—C7—C12—C19	103.4 (3)	C20—C19—C26—C27	−175.0 (2)
C8—C7—C12—C13	154.0 (2)	C12—C19—C26—C27	1.5 (4)
C5—C7—C12—C13	−26.1 (3)	C28—O3—C27—O4	0.4 (3)
C7—C12—C13—C14	−74.5 (3)	C28—O3—C27—C26	179.4 (2)
C19—C12—C13—C14	155.7 (2)	N2—C26—C27—O4	−3.8 (4)
C7—C12—C13—C18	103.5 (2)	C19—C26—C27—O4	171.9 (3)
C19—C12—C13—C18	−26.2 (3)	N2—C26—C27—O3	177.19 (19)
C18—C13—C14—C15	−2.5 (4)	C19—C26—C27—O3	−7.1 (4)
C12—C13—C14—C15	175.6 (2)	C27—O3—C28—C29	179.4 (2)

### Hydrogen-bond geometry (Å, º)

**Table:**

*D*—H···*A*	*D*—H	H···*A*	*D*···*A*	*D*—H···*A*
N1—H1*A*···O2^i^	0.86	2.21	2.955 (3)	144
N2—H2*A*···O4^ii^	0.86	2.07	2.880 (3)	157

Symmetry codes: (i) −*x*, −*y*+2, −*z*+1; (ii) −*x*+1, −*y*+1, −*z*+2.
